# Disrupted Homeostatic Cytokines Expression in Secondary Lymph Organs during HIV Infection

**DOI:** 10.3390/ijms17030413

**Published:** 2016-03-22

**Authors:** Lintao Zhao, Jianbao Gao, Yan Li, Lina Liu, Yang Yang, Bo Guo, Bo Zhu

**Affiliations:** 1 Department of Oncology, Xinqiao Hospital, Third Military Medical University, Chongqing 400037, China; chaoren72@126.com (L.Z.); nwgjb@163.com (J.G.); lln372@126.com (L.L.); yangoncology@163.com (Y.Y.); 2 Department of Nephrology, Xinqiao Hospital, Third Military Medical University, Chongqing 400037, China; xili72@163.com; 3 Department of Pathogenic Biology, Third Military Medical University, Chongqing 400037, China

**Keywords:** human immunodeficiency virus (HIV) infection, secondary lymph organs, CCL21, IL-7

## Abstract

Research has firmly established that infection by human immunodeficiency virus (HIV) leads to structural disruption in secondary lymph organs (SLOs) and that IL-7 expression by SLOs is downregulated in simian immunodeficiency virus (SIV)-infected rhesus macaques. However, the foregoing has not been demonstrated in HIV-infected patients. As well, SLO-produced chemokines and cytokines, other than IL-7, have not been tested. In this study, SLOs in HIV-infected patients exhibit decreased levels of lymphoid cytokines, such as IL-7 and C–C motif chemokine ligand 21 (CCL21), due to lower expression of lymphotoxin (LT)-β. Previous research has shown that LT-β is produced mainly by CD4^+^T cells in rhesus macaques, while our study found the same level of LT-β expressed by CD4^+^T and CD8^+^T cells in humans. CD8^+^T cells substitute for depleted CD4^+^T cells LT-β production. Only the total number of CD3^+^T cells can account for the majority of LT-β in human SLOs. This study indicates a possible mechanism and a potential target for improvement of SLO function in HIV-infected patients, a novel adjuvant therapy for AIDS.

## 1. Introduction

Mortality due to human immunodeficiency virus (HIV) infection can be mitigated by suppressing HIV replication and by restoring CD4^+^T cell populations. However, control of viral replication and restoration of CD4^+^T cells population fails to fully reconstitute the immune system [1,2]. Importantly, even the peripheral blood population of CD4^+^T cells recovers modestly, while HIV remarkably reduces their numbers in secondary lymph organs (SLOs) where T cells reside, such as the spleen, lymph nodes, and mucosal lymphoid tissues [3,4]. Reconstruction of the disrupted SLO structures requires more time than some other tissues.

According to previous research on rhesus macaques infected with pathogenic simian immunodeficiency virus (SIV), lymphotoxin (LT)-β derived from CD4^+^T cells plays a key maintenance role in lymph node structure, especially in regard to networks of fibroblastic reticular cells (FRCs) and follicular dendritic cells (FDCs) [5]. Depletion of naive CD4^+^T cells leads thereby to damaged SLO structures, which recover both slowly and incompletely [6]. The preceding findings explain why even restoration of CD4^+^T cell population and control of viral replication may fail to fully reconstitute the immune system [7].

Generation of immune response requires rare, antigen-specific T cells to encounter dendritic cells (DCs) that present the appropriate antigen. Such a spontaneous encounter is rare *in vivo,* and only occurs in SLOs. SLOs contain several compartments that are characterized by specific resident stromal cells [8,9,10], of which the most important are the B- and T-cell zones. The B-cell zone is composed of FDCs, which produce C–X–C motif chemokine 13 (CXCL13) to attract B cells [11], and the T-cell zone is rich in FRCs expressing many T-cell response-modulating cytokines, such as C–C motif chemokine ligand 19 (CCL19), CCL20, IL-7, and CCL21 [9]. As well, FRCs are able to secrete extracellular matrices (ECMs) abundantly, thus forming special conduits that transport small molecules to the T-cell zone [10,11].

Moreover, FRCs are the major source of IL-7, responsible for homeostatic proliferation of T cells, reduced expression of which interleukin has been found in SIV-infected rhesus macaque SLOs, though CCL21 and other chemokines, also mainly produced by FRCs, have not been tested. However, previous research indicates that these cytokines also play an important T cell response regulatory role. For example, less CCL21 in lymphoid tissues inhibits the aggregation of T cells and DCs in the T-cell zone of SLOs, thereby lowering the probability of encounter between antigen-specific T cells and DCs, and weakening the intensity of immune response [12,13].

As reported earlier of the lymphoid stromal network, LT-β induces production by FRCs of many cytokines, such as IL-7 and CCL21 [14,15,16,17,18]. The ablation of FRC-specific LT-β receptors profoundly abolishes the expression by FRCs of such molecules as CCL21 and IL-7 [19]. These findings arouse our interest in the effect of CD4^+^T cells deletion on expression of LT-β, thus influencing other cytokines secreted by FRCs in lymph nodes.

In this study, we found that lymphoid cytokines are downregulated in SLOs of HIV-infected patients and which change correlates with LT-β level. Moreover, CD4^+^T and CD8^+^T cells exhibit no difference in LT-β expression. CD8^+^T cells can substitute for depleted CD4^+^T cells in LT-β production. Reduced LT-β only appeared with a decreased number of CD3^+^T cells.

## 2. Results

### 2.1. Different Cytokines were Downregulated in Secondary Lymph Organs (SLOs) of Human Immunodeficiency Virus (HIV)-Infected Patients

**Figure 1:**
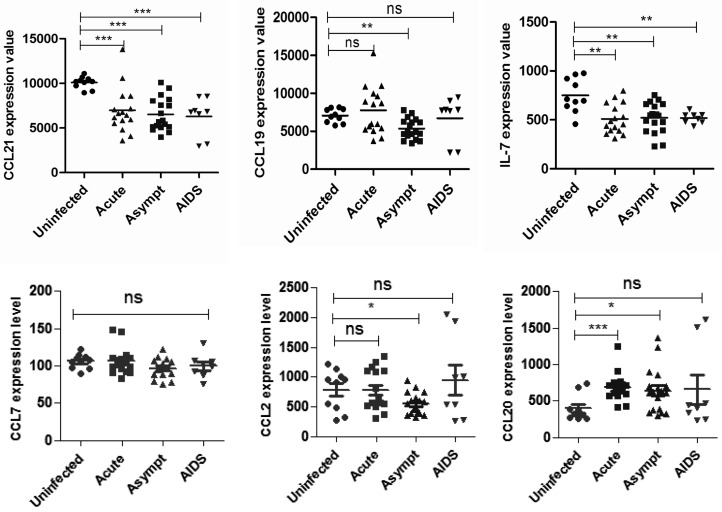
Lymphoid cytokine downregulation in human immunodeficiency virus (HIV)-infected patients. Microarray analysis of HIV-infected patients: mRNA expression of cytokines in lymph nodes at different stages (uninfected, acute, asymptomatic, or AIDS stage).* *p* < 0.05; ** *p* < 0.01; *** *p* < 0.001; ns indicates not significant.

Lowered IL-7 expression in SLOs has been reported in SIV-infected rhesus macaques [5,6]. Searching the Gene Expression Omnibus (GEO) DataSets, we found a relevant study published on 2 June 2009 [20]. On the basis of this study, we analyzed inguinal lymph nodes from 5 uninfected individuals and 22 untreated HIV-infected individuals at different clinical stages: 9 patients with acute stages of AIDS, 9 patients with asymptomatic stages of AIDS, and 4 patients with AIDS. When compared with uninfected individuals, HIV-infected patients of varying clinical stages showed significant decline in lymph nodes IL-7 levels. Similar to variation trends for IL-7, CCL21 expression decreased obviously at different stages of HIV infection. We also checked the expression of other cytokines associated with T cell immune response, such as CCL7, CCL2, and CCL20, which did not vary significantly (Figure 1).

### 2.2. Lymphotoxin (LT)-β Plays a Critical Role in Regulating C–C Motif Chemokine Ligand (CCL12) and IL-7 Expression in Lymph Nodes of HIV-Infected Patients

**Figure 2:**
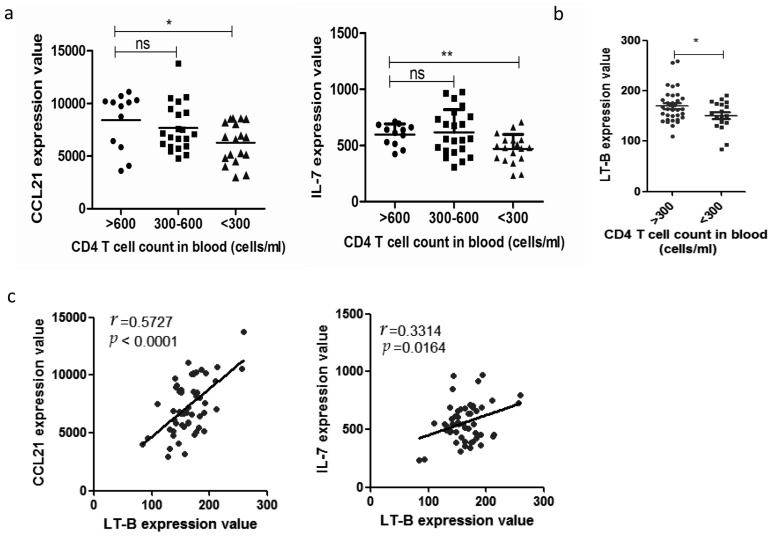
Decreased expression of C–C motif chemokine ligand 21 (CCL21) and IL7 as caused by reduced expression of lymphotoxin (LT)-β in lymph nodes: (**a**) microarray analysis of HIV-infected patients, mRNA expression levels of CCL21, and IL-7 in lymph nodes according to CD4^+^T cell numbers in peripheral blood; (**b**) analysis HIV-infected patients, expressions of LT-β mRNA in lymph nodes according to CD4^+^T cell numbers in peripheral blood; (**c**) lymph nodes of HIV-infected patients with mRNA expression of CCL21 and IL-7 correlated to mRNA expression of LT-β in lymph nodes. * *p* < 0.05; ** *p* < 0.01, ns indicates not significant.

HIV infection in humans is characterized by sustained depletion of naive CD4^+^T cells. Re-analysis of the GEO DataSet, GSE16363 revealed a statistically significant descending trend in expression of CCL21 and IL7 (Figure 2a) once CD4^+^T cell numbers dropped below 300 cells/mL, which decline accompanied with the number of CD4^+^T cells in an individual’s peripheral blood. CD4^+^T cells produce LT-β in rhesus macaques, while LT-β is critical for SLOs to generate CCL21 and IL-7 [17,18]. Our analysis indicates that CD4^+^T cell depletion is accompanied by a significant decrease in LT-β (Figure 2b). In addition, LT-β correlates significantly with CCL21 and IL-7 expression (Figure 2c).

### 2.3. Absence of CD4^+^T cells Have Little Effect on Cytokines Expression in SLOs

**Figure 3:**
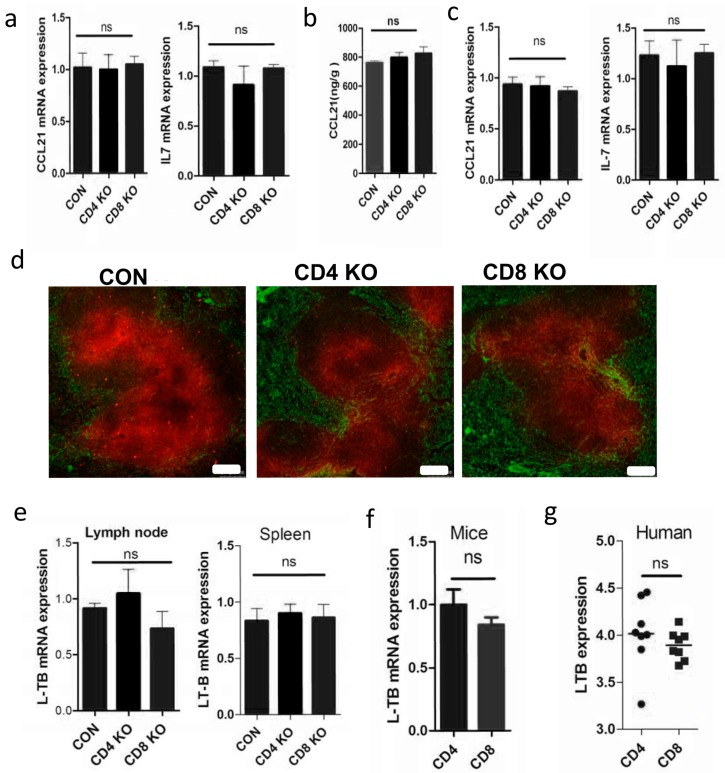
Lymph cytokines are not significantly different without CD4^+^T cells. (**a**) Cytokine expression in lymph nodes of wild-type, knockout mice lacking CD4^+^T or CD8^+^T cells (CD4KO or CD8KO) as quantified by RT-qPCR and (**b**) detected by ELISA in tissue homogenates; (**c**) cytokine expression in spleens of wild-type, CD4KO, and CD8KO mice as quantified by RT-qPCR; (**d**) spleen cryostat sections were stained for CCL21 (red) and ER-TR7 (green; scale bar: 75 µm); (**e**) LT-β expression in lymph nodes and spleens of wild-type, CD4KO, and CD8KO mice; (**f**) mouse CD4^+^ and CD8^+^T cells as determined by RT-qPCR; and (**g**) LT-β expression as determined by microarray analysis of circulating CD4^+^ and CD8^+^T cells as isolated from eight healthy people. ns indicates not significant.

As reported, LT-β is mainly expressed by CD4^+^T cells in rhesus macaques, where it maintains expression of CCL21 and IL-7 in the lymph nodes. To verify the relationship between T cells and cytokine expressions, we used knockout mice lacking CD4^+^T or CD8^+^T cells (CD4KO or CD8KO). Surprisingly, lymph node mRNA expression levels of CCL21 and IL-7 show no difference between controls and KO mice (Figure 3a), as is further confirmed by ELISA detection of CCL21 (Figure 3b). Besides lymph nodes, the spleen exhibits similar mRNA expression patterns for CCL21 and IL-7 (Figure 3c), confirmed again by immunofluorescence staining of CCL21 (Figure 3d). In addition, LT-β also exhibits no change in SLOs of CD4KO or CD8KO mice (Figure 3e). As well, CD4^+^T cells and CD8^+^T cells sorted from C57BL/6 mice exhibit a similar LT-β mRNA expression level (Figure 3f). Human gene expression profiling by array in the GEO DataSets (GSE31773) also supported a similar LT-β mRNA expression level between CD4^+^T cells and CD8^+^T cells (Figure 3g). Taken together, the above results suggest that disrupted expression of cytokines in SLOs is not explained entirely by the depletion of CD4^+^T cells in HIV-infected patients. We speculated that CD8^+^T cells may compensate for LT-β expression after depletion of CD4^+^T cells.

### 2.4. Elimination of Total T Cells Reduced LT-β Expression in SLOs

**Figure 4:**
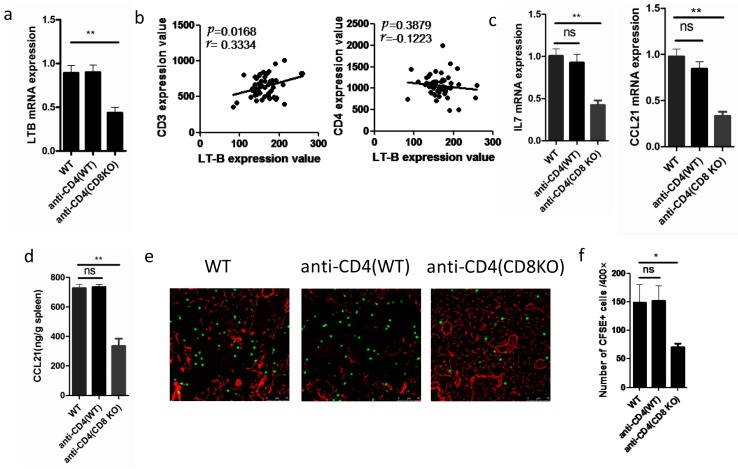
Lymph cytokines are significantly different without T cells. (**a**) LT-β mRNA expressions in the lymph nodes of wild-type, anti-CD4 (wild-type), or anti-CD4 (CD8KO) mice were quantified by RT-qPCR; (**b**) in microarray analysis of HIV-infected patients, mRNA expression of LT-β correlates with mRNA expression of CD3 in lymph nodes, but not CD4; (**c**) cytokines expressions in the lymph nodes of wild-type, anti-CD4 (wild-type), or anti-CD4 (CD8KO) mice were quantified by RT-qPCR and (**d**) detected by ELISA in tissue homogenates; (**e**) naive CD8^+^T lymphocytes were purified, labeled with carboxyfluorescein diacetate succinimidyl ester (CFSE), and transfected to wild-type, anti-CD4 (wild-type), or anti-CD4 (CD8KO) mice, and their localization in lymph nodes was ascertained 6 h later. Lymph node cryostat sections were stained for CFSE (green) and ER-TR7 (red; scale bar: 75 µm); (**f**) the numbers of CFSE ^+^ cells present in lymph nodes of wild-type, anti-CD4 (wild-type), or anti-CD4 (CD8KO) mice. * *p* < 0.05; ** *p* < 0.01, ns indicates not significant.

As CD4^+^T and CD8^+^T cells contribute equally to the production of LT-β, we wondered whether LT-β expression levels could be affected by the total T cell number. As expected, the level of LT-β was lower when CD4^+^T cells were abolished by CD4 antibody in CD8KO mice (Figure 4a), which suggests that CD8^+^T cells compensate for the LT-β expression of deleted CD4^+^T cells. Moreover, LT-β expression correlates with CD3 mRNA expression value, but not CD4, in lymph nodes of HIV-infected patients (Figure 4b), a result that further proves that lower LT-β level is caused by a decreased total number of CD3^+^T cells.

### 2.5. Depletion of Total T Cells Disrupts Cytokine Expression in SLOs

In SLOs, LT-β is critical for inducing production of IL-7 and CCL21 [14,15,16,17,18]. Consequently, CCL21 and IL7 decreased in spleen tissue after the depletion of CD4^+^T cells in CD8KO mice (Figure 4c,d). CCL21 is known to regulate migration of naive T cells in SLOs [8,21]. Hence, we transferred naive carboxyfluorescein diacetate succinimidyl ester (CFSE)-labeled CD8^+^T cells into WT, anti-CD4-WT, and anti-CD4-CD8KO mice. Weakened migration ability was observed in lymph nodes of anti CD4-CD8KO mice (Figure 4e,f).

## 3. Discussion

Decreased CD4^+^T cell count due to HIV infection results in the downregulation of IL-7, plus structural changes in SLOs, affecting the survival and activation of T cells [22,23,24]. Despite the efficient control of viral populations by antiviral treatment, full immune response is not recovered *in vivo* even when normal peripheral CD4^+^T cell count is restored due to slow repair mechanisms in SLOs [1,3,5].

SLOs play an important role in regulating T cell response, mainly through FRCs [25]. FRCs secrete copious cytokines, which are critical for hematopoietic cell recruitment and their localization within SLOs. For example, CCL2 and CCL7 facilitate the recruitment of receptor-expressing memory T cells and DCs. The lymphocyte chemo-attractants, CXCL12, and CCL20, promote egress of activated T cells from lymph nodes. IL-7 is responsible for homeostatic proliferation of T cells [26]. CCL21 recruits antigen-specific T cells with DCs to SLOs. Tests on the expression of other cytokines associated with T cells immune response, such as CCL7, CCL2, CCL20, CCL21, and IL7, show that IL7 and CCL21 expression decreases markedly at different stages of HIV infection. Such results suggest that the microenvironment in secondary lymph organs (SLOs) is disrupted during HIV infection. Indeed, many studies have shown that plasma levels of IL-7 actually increase in advanced HIV-1 infection. However, our results show that levels of IL7 and CCL21 actually decrease in lymph nodes under advanced HIV-1 infection, which is consistent with our perspective that peripheral recovery of CD4+ T cells does not represent their recovery in lymph nodes.

In rhesus macaques, LT-β is reported to be expressed mainly by CD4^+^T cells [5]. Moreover, LT-β has been confirmed to maintain SLO expression of CCL21, IL7, and many other cytokines in SLOs. Thus, we hypothesized that HIV infection reduces CD4^+^T cells, thereby also reducing LT-β level. Our results provide proof of the effects on expression of various cytokines in the lymph nodes of HIV patients, especially LT-β-correlated CCL21 and IL7. However, results for LT-β levels remained unchanged in CD4KO mice. Considering developmental differences in CD4KO mice, tests were performed on depletion of CD4^+^T cells in wild-type mice with the same results. This study finds no differences in LT-β expressions between CD4^+^T and CD8^+^T cells in humans and mice, which is inconsistent with results for rhesus macaques. Together, the above investigation suggests that CD8^+^T cells replace LT-β secretion lacking from apoptotic CD4^+^T cells in HIV-infected patients.

We further find reduced cytokine expression levels in HIV patients in the absence of decreased total CD3^+^T cells. As well, microarray data for lymph nodes of HIV-infected patients reveal a correlation between LT-β expression level and CD3, but no correlation with CD4. The changing trends of CD4^+^T and CD8^+^T counts vary among HIV patients. There are many factors contributing to reduced CD8^+^T counts, which do not always accompany the loss of CD4^+^T cells. Our results show that the level of lymphotoxin β correlates with CD3, but not with CD4, a result that is highly consistent with our previous results. Lastly, the migration of naive T cells in SLOs, controlled by CCL21 [8,21], is attenuated by low expression. All of the preceding factors contribute to a deteriorated immune response by HIV-infected patients.

## 4. Materials and Methods

### 4.1. Ethics Statement

All animal experiments herein were approved by the Animal Ethical and Experimental Committee of the Third Military Medical University. All animal surgery was performed under sodium pentobarbital anesthesia, and all efforts were made to minimize suffering.

### 4.2. Mice

Eight-week-old female C57BL/6 (control), CD4-knock-out (CD4KO), and CD8-knock-out (CD8KO) mice were purchased from the Center of Experimental Animals at Third Military Medical University (TMMU, Chongqing, China). The mice were raised according to TMMU Guidelines of Animal Experiments. Mice were randomly divided into different groups. For antibody treatment *in vivo*, the mice were injected with Ab anti-mouse CD4 (500 µg/mouse, clone GK1.5). At 4 days after Ab-treatment, the depletion of individual cell types was assessed as almost complete (>99%), according to flow cytometry. All animal experimental protocols used in this study are in accordance with institutional Guidelines for Animal Experiments and were approved by Institutional Ethics Committee of the TMMU.

### 4.3. Cell Migration in Vivo

To test cell migration *in vivo*, naive splenocytes were purified using anti-CD8 magnetic beads in accordance with the manufacturer’s instructions. The purified lymphocytes were labeled with 4 μm CFSE (Molecular Probes, Eugene, OR, USA), and then 5 × 10^6^ cells were transferred into the recipient mice [27]. After 6 h, the mice were euthanized, whose lymph nodes were then removed for microscopic analysis.

### 4.4. Immunofluorescence Microscopy

Subject spleen and lymph nodes were frozen in optimal cutting temperature compound (OCT) (TissueTek, Elkhart, IN, USA). Cryostat sections (10 μm) were fixed in ice-cold acetone for 10 min, and then stained with an anti-mouse ER-TR7 antibody (Santa Cruz Biotechnology Inc., Santa Cruz, CA, USA) and an anti-mouse CCL21 antibody (R & D Systems, Minneapolis, MN, USA)[12]. The primary antibodies were detected using fluorescein isothiocyanate (FITC)-conjugated anti-rat (1:100, Beyotime Institute of Biotechnology, Beijing, China) or Cy3-conjugated anti-goat IgG (1:200, Beyotime).

### 4.5. Enzyme-Linked Immunosorbent Assay (ELISA)

Tissues were removed from the mice and placed in a phosphate buffer solution (PBS) supplemented with 1% bovine serum albumin and 1 mM of pheylmethylsulfonyl fluoride (PMSF, Beyotime, Guangzhou, China). After the tissues were homogenized, CCL21 and CCL19 were detected using a DuoSet ELISA development kit (R & D Systems) per the manufacturer’s instructions [12].

### 4.6. Real-Time Quantitative Reverse Transcription Polymerase Chain Reaction (RT-qPCR)

Encoding of PCR primer pairs, including their specificity, orientation (F: forward, R: reverse) and sequence were as follows: CCL21 (F: CCCTGGACCCAAGGCAGT, R: GGCTTAGAGTGCTTCCGGG), actin (F: CCTGAGGCTCTTTTCCAGCC, R: AGAGGTCTTTACGGATGTCAACGT) [15], and IL-7 (F: GTGCCACATTAAAGACAAAGAAG, R: GTTCATTATTCGGGCAATTACTATC). According to the manufacturer’s instructions, whole spleens or lymph nodes were placed in Trizol (Invitrogen Life Technologies, Carlsbad, CA, USA), and total RNA was extracted. Total chemokine mRNA was detected via RT-qPCR using SYBR Green (Takara Bio Inc., Tokyo, Japan) with a BioRad (Hercules, CA, USA) RT-qPCR analyzer. The conditions were set as follows: 95 °C for 30 s, followed by 40 cycles of 95 °C for 5 s, 59 °C for 34 s, 95 °C for 15 s, 60 °C for 60 s, and 95 °C for 15 s. Expression was quantified via 2^-ΔΔCt^ with actin as a reference.

### 4.7. Availability of Supporting Data

The data sets supporting results in this study are available in the GEO DataSets repository: GSE16363 at http://www.ncbi.nlm.nih.gov/geo/query/acc.cgi?acc=GSE16363. Inguinal lymph nodes from 5 uninfected individuals and 22 untreated, HIV-infected individuals at different clinical stages were analyzed in this datasets: 9 patients with acute stages of AIDS, 9 patients with asymptomatic stages of AIDS, and 4 patients with AIDS. Each sample was duplicated using microarray hybridization [20,28,29,30]. GSE31773 mRNA samples were collected from circulating CD4^+^T and CD8^+^T cells in eight healthy donors. GSE31773 is available at http://www.ncbi.nlm.nih.gov/ geo/query/acc.cgi?acc= GSE31773.

### 4.8. Statistical Analysis

Statistical analysis was performed by two-tailed, unpaired *t*-test on Prism 5.01 (GraphPad Software Inc, La Jolla, CA, USA). Each group contained *n* = 5 mice, while differences with *p* values of less than 0.05 were treated as statistically significant (* *p* < 0.05; ** *p* < 0.01, ns indicates not significant). Linear regression analysis was performed using Prism 5.0 wells.

## 5. Conclusions

In general, our results further validate that HIV infection can significantly destroy SLO functionality. As well, improvement of lymph node function could be a new adjuvant therapy for AIDS.

## Abbreviations

HIV: human immunodeficiency virus
SLOs: secondary lymph organs
LT-β: lymphotoxin (LT)-β
FRCs: fibroblastic reticular cells
FDCs: follicular dendritic cells
DCs: dendritic cells
CD4KO or CD8KO: CD4^+^T or CD8^+^T cells knockout
